# Autophagic Inhibition via Lysosomal Integrity Dysfunction Leads to Antitumor Activity in Glioma Treatment

**DOI:** 10.3390/cancers12030543

**Published:** 2020-02-27

**Authors:** Hui-Yun Hwang, Yoon Sun Cho, Jin Young Kim, Ki Na Yun, Jong Shin Yoo, Eunhyeong Lee, Injune Kim, Ho Jeong Kwon

**Affiliations:** 1 Chemical Genomics Global Research Laboratory, Department of Biotechnology, College of Life Science and Biotechnology, Yonsei University, Seoul 03722, Korea; ghkdgmldbs@naver.com (H.-Y.H.); yoonycoo@gmail.com (Y.S.C.); 2 Biomedical Omics Group, Korea Basic Science Institute, Ochang, Chungbuk 28119, Korea; jinyoung@kbsi.re.kr (J.Y.K.); 714kina@kbsi.re.kr (K.N.Y.); jongshin@kbsi.re.kr (J.S.Y.); 3 Graduate School of Medical Science and Engineering, Korea Advanced Institute of Science and Technology, Daejeon 34141, Korea; leh0617@kaist.ac.kr (E.L.); injunek@kaist.ac.kr (I.K.)

**Keywords:** autophagy, autophagonizer, target identification of label-free compound, target validation, autophagic flux, autophagy inhibition, lysosomal integrity function of Hsp70

## Abstract

Manipulating autophagy is a promising strategy for treating cancer as several autophagy inhibitors are shown to induce autophagic cell death. One of these, autophagonizer (APZ), induces apoptosis-independent cell death by binding an unknown target via an unknown mechanism. To identify APZ targets, we used a label-free drug affinity responsive target stability (DARTS) approach with a liquid chromatography/tandem mass spectrometry (LC–MS/MS) readout. Of 35 protein interactors, we identified Hsp70 as a key target protein of unmodified APZ in autophagy. Either APZ treatment or Hsp70 inhibition attenuates integrity of lysosomes, which leads to autophagic cell death exhibiting an excellent synergism with a clinical drug, temozolomide, in vitro, in vivo, and orthotropic glioma xenograft model. These findings demonstrate the potential of APZ to induce autophagic cell death and its development to combinational chemotherapeutic agent for glioma treatment. Collectively, our study demonstrated that APZ, a new autophagy inhibitor, can be used as a potent antitumor drug candidate to get over unassailable glioma and revealed a novel function of Hsp70 in lysosomal integrity regulation of autophagy.

## 1. Introduction

Autophagic cell death is a type of apoptosis-independent cell death without chromatin condensation. It is accompanied by bulk-scale autophagic vacuolization, autophagosomes formation leading to fusion with lysosomes to form autolysosomes in the cytoplasm [1,2]. Therefore, the maintenance of intact lysosomal integrity is pivotal to degrade the cellular components through sequential events of autophagy. Autophagy plays a crucial role in modulating cancer cellular homeostasis [3]. Targeting autophagy in cancer has provided opportunities for drug development [4,5], but autophagy modulation in cancer either promotes tumor suppression or survival according to specific autophagy pathways [6]. Hence, understanding these differences and the exact biological mechanism of autophagy is of fundamental and therapeutic importance. Recent studies identified a number of inhibitory small molecules of autophagy that effectively target cancer [7,8,9], demonstrating the potential value of these compounds for cancer therapeutics. This strategy has been a focus of anticancer drug research, yet few candidates have shown this property or developed it as effective anticancer compounds.

Previously, we identified a novel autophagy regulator autophagonizer (APZ) that induces autophagosome (AP)-like vesicles and induces apoptosis-independent cancer cell death [10]; therefore, by inducing autophagic cell death through large-scale autophagic vacuolization, APZ has potential for development as a novel autophagy inhibitor for anticancer therapy. Our previous study demonstrated that APZ induces large-scale accumulation of autophagosomes in the cytoplasm and triggers apoptosis-independent autophagic cell death. However, the cellular targets of APZ and the mechanism by which it mediates autophagic cell death remain unknown. Clarification of these issues will provide new routes and targets for cancer therapy.

To explore the molecular mechanism underlying the autophagy-regulating activity of APZ, DARTS and LC–MS/MS analysis were applied to identify the target proteins of APZ in its native form without chemical modification. We recently presented potent effectiveness for the label-free target identification method, DARTS and LC–MS/MS, by determining protein target and validating its biological relevancy of clinically well-known immunosuppressive drug, FK506, as a new autophagy flux inducer by binding V-ATPase [11]. In this study, the DARTS and LC–MS/MS method was expansively applied to identify a biologically relevant protein target of target unknown small molecule, APZ, and investigated its potential as a new anticancer agent. Herein, we report that APZ antagonizes Hsp70, inducing lysosomal dysfunction, inhibiting autophagic flux, which is called to impaired autophagy that might be caused by inhibiting lysosomal integrity, and ultimately leading to cell death in cancer cells and tumors. Notably, we reveal a novel function of Hsp70 in autophagy in the step of lysosome integrity into autophagosome. Collectively, APZ is a new small molecule for inducing autophagic cell death with potential as an anticancer agent for glioma treatment.

## 2. Results

### 2.1. Autophagonizer Functions Not by Inducing Autophagy, but by Inhibiting Autophagy

**Figure 1:**
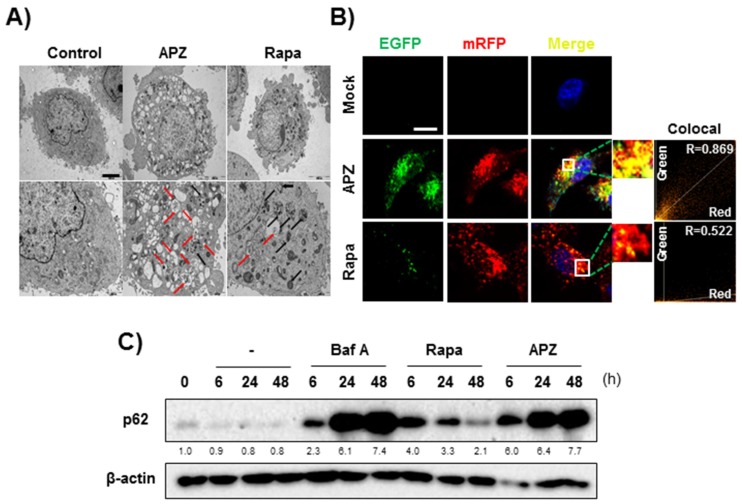
Autophagonizer (APZ) inhibits autophagic flux. (**A**) TEM images of APZ (5 μM)- and rapamycin (10 μM)-induced autophagic vacuoles after 24 h of treatment. Red arrows indicate autophagosomes containing multiple double-membrane structures. Black arrows indicate autolysosomes containing single-membrane, multilamellar structures. Scale bar, 10 μm. (**B**) Autophagic flux evaluation in human cervical cancer (HeLa) cells using monomeric red fluorescent protein (mCherry)-green fluorescent protein (GFP) LC3in the presence of each compound. Representative images of merged channels are shown; scale bar, 10 μm. Pearson coefficient for the colocalization analysis is shown. (**C**) p62 levels after compound treatment for 6, 24, and 48 h. APZ induced accumulation of p62 for up to 48 h, similar to bafilomycin A (10 nM). More details of western blot, please view at the supplementary materials.

APZ was recently discovered as an autophagy regulator by a phenotype-based small molecule screen [10]. APZ induces autophagy-dependent cell death in various cancer cell lines, including HeLa and HT1080. However, the mechanism responsible for its biological activity remained unknown. To explore the underlying mechanism of APZ in autophagy-dependent cell death, we first investigated autophagy in cells treated with APZ by transmission electron microscopy (TEM) (Figure 1A). Notably, APZ-treated cells exhibited significant accumulation of autophagic vesicles, particularly autophagosomes containing multiple double-membrane structures (red arrows). In contrast, rapamycin, a well-known autophagy inducer, resulted in a smaller number of these structures and many more autolysosomes containing single-membrane and multilamellar structures (black arrows), indicating a different mode of action of APZ from that of rapamycin. Next, the effect of APZ on autophagic flux was investigated using double fluorescent-tagged chain protein type 3 (LC3) (mRFP/mCherry-GFP) to visualize autophagic flux based on the differing pH stabilities of GFP and mRFP. GFP confers weak stability and is degraded in acidic environments, such as lysosomes and autolysosomes. Therefore, if green/red (yellow) fluorescence accumulates in intracellular compartments, incomplete autophagy occurs through disruption of lysosome integrity or fusion of autophagosomes with lysosomes. Unexpectedly, APZ treatment resulted in yellow fluorescence accumulation, indicative of autophagosomes, leading to incomplete autophagy (Figure 1B). In contrast, rapamycin treatment induced red fluorescence accumulation in HeLa cells. Thus, APZ inhibits rather than induces autophagy, resulting in abnormal, impaired autophagy.

P62 is a protein expressed in late macro-autophagy and degraded in response to increasing autophagic flux, allowing its use to assess this flux [12]. As shown in Figure 1C, p62 levels increased for 48 h after APZ treatment, whereas the levels decreased from 24–48 h after rapamycin treatment. In addition, the levels of essential early markers for autophagosome formation, Atg5 and Atg16L [13], were dose-dependently reduced by APZ treatment, whereas p62 and the lipid conjugated form of LC3 (LC3-II) gradually accumulated (Figure S1). Hence, unlike rapamycin, APZ inhibits autophagic flux suggesting it promotes nonapoptotic cell death by the induction of autophagic cell death.

### 2.2. APZ Induces Autophagic Cell Death in Cancer Cell Lines

**Figure 2:**
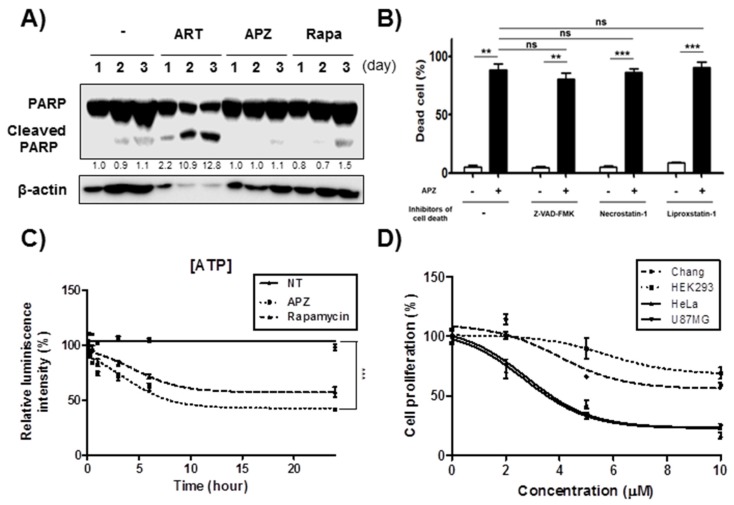
APZ triggers cancer-specific autophagic cell death. (**A**) Treatment with APZ (5 μM) did not induce poly (ADP-ribose) polymerase (PARP) cleavage in HeLa cells for 24 or 72 h of treatment. Artesunate (ART, 25 μM) was used as a positive control for apoptosis. (**B**) Effect of various inhibitors on cell death induced by APZ treatment. Cells were pretreated with DMSO (control) or the inhibitors of cell death, Z-VAD-FMK (50 μM), necrostatin-1 (10 μM), and liproxstatin-1 (100 nM) for 1 h before treatment with APZ (10 μM) for 24 h. The percentage of nonviable cells was assessed by trypan blue staining. Values represent mean ± SEM from three independent experiments. (**C**) Adenosine triphosphate (ATP) levels in HeLa cells were measured using an ATPlite luminescence assay system. ATP levels decreased following treatment with APZ (5 μM) or rapamycin (10 μM) for 24 h. Values are means ± SEM; *n* = 3. **p < 0.01, ***p < 0.001. (**D**) Effect of APZ on the proliferation of normal cell lines, including Chang and human embryonic kidney (HEK293), and cancer cell lines, including HeLa and U87MG. All cells were treated with APZ (1–10 μM) for 48 h, and cell growth was measured using an 3-(4,5-dimethylthiazol-2-yl)-2,5-diphenyl tetrazolium bromide (MTT) colorimetric assay. *N* = 4 means ± SE. More details of western blot, please view at the supplementary materials.

In the previous study, we showed that APZ treatment induces apoptosis-independent cell death in several cancer cell lines for 24 h [10]. Artesunate [14] (ART) induces apoptosis with characteristic by activating PARP (Poly (ADP-ribose) polymerase) cleavages [15]. Here, we investigated further whether APZ induces apoptosis after a long period of treatment from 24–72 h, because autophagy process can precede G2/M cell cycle arrest and apoptosis intervenes later to trigger apoptotic cell death [16]. Notably, APZ neither induced PARP cleavage (Figure 2A) nor induced apoptosis. The effect of various inhibitors on cell death induced by APZ treatment was also investigated. The inhibitors of cell death Z-VAD-FMK (apoptosis inhibitor, 50 μM), necrostatin-1 (necrosis inhibitor, 10 μM), and liproxstatin-1 (ferroptosis inhibitor, 100 nM) was pretreated for 1 h and then APZ (10 μM) was treated for 24 h. Interestingly, the percentage of nonviable cells was not decreased in APZ-treated cultures (Figure 2B), implying that APZ induced autophagic cell death independent from apoptosis, necrosis, and ferroptosis, as well. To further elucidate the type of cell death induced by APZ, ATP levels were measured upon APZ treatment. APZ treatment gradually reduced ATP levels in the cells from as early as 5 min to 24 h, similar to rapamycin, which inhibits glycolysis to generate ATP [17] (Figure 2C). In normal cell lines, including Chang and HEK293 cells, treatment with APZ for 48 h showed differential sensitivity in cell proliferation and mitochondrial activity assays (Figure 2D). Cell proliferation remained greater than 50% at 48 h in normal cell lines even with a high dose of APZ (10 μM), whereas HeLa and other cancer cell lines exhibited IC_50_ values below 5 μM. Hence, cancer cell lines (HeLa, U87MG) are more sensitive to APZ than those of normal cell lines probably due to activation of autophagy-induced cell death by APZ.

The glioblastoma cell line U87MG was also sensitive to APZ in vitro as determined by the MTT assay (Figure 2D). Therefore, these cells were used in a glioblastoma mouse xenograft model (Figure S2A). Notably, APZ exhibited antitumor activity by decreasing tumor volume (vehicle-treated group: 1547 mm^3^ [100%], APZ-treated group: 702 mm^3^ [45%], 18 days) with no toxicity towards mice over the 18-days treatment. Acridine orange staining of APZ-treated xenograft tumors (Figure S2B) revealed that the red fluorescence indicative of lysosomal integrity was significantly reduced by APZ treatment. These results demonstrated that APZ could inhibit lysosomal integrity and proteolysis, which lead to impaired autophagy resulting in autophagic cell death of xenograft tumors.

The low sensitivity to chemotherapeutic agent temozolomide (TMZ) of U87MG cells in vitro has been considered as one of the poor prognosis of patients despite of the addition of TMZ to radiation [18]. Therefore, there has been urgent demand for the development of adjuvant or synergic agent with TMZ. Cell proliferation of U87MG cells was inhibited by APZ treatment with IC_50_ values below 5 μM, whereas TMZ did not exhibit the remarkable anticancer effect at 72 h even with a high dose of 300 μM (Figure S3A). Interestingly, however, APZ exhibited significant synergism with TMZ over 72 h (Figure S3B) validating from the combination index (CI) method [19]. In this analysis, a value of <1 indicates synergism and the lowest CI value for APZ in combinational effect with TMZ in U87MG was 0.35 (Figure S3C). To further investigate the effectiveness and the safety of APZ as a chemotherapeutic agent in vivo, a mouse orthotopic glioma model was used by intracranial injecting GFP-GL261 mouse glioma cells into congenic B6 wild type mice. GL216 glioma was known to exhibit the malignant characteristics in high-grade glioma [20,21].

**Figure 3:**
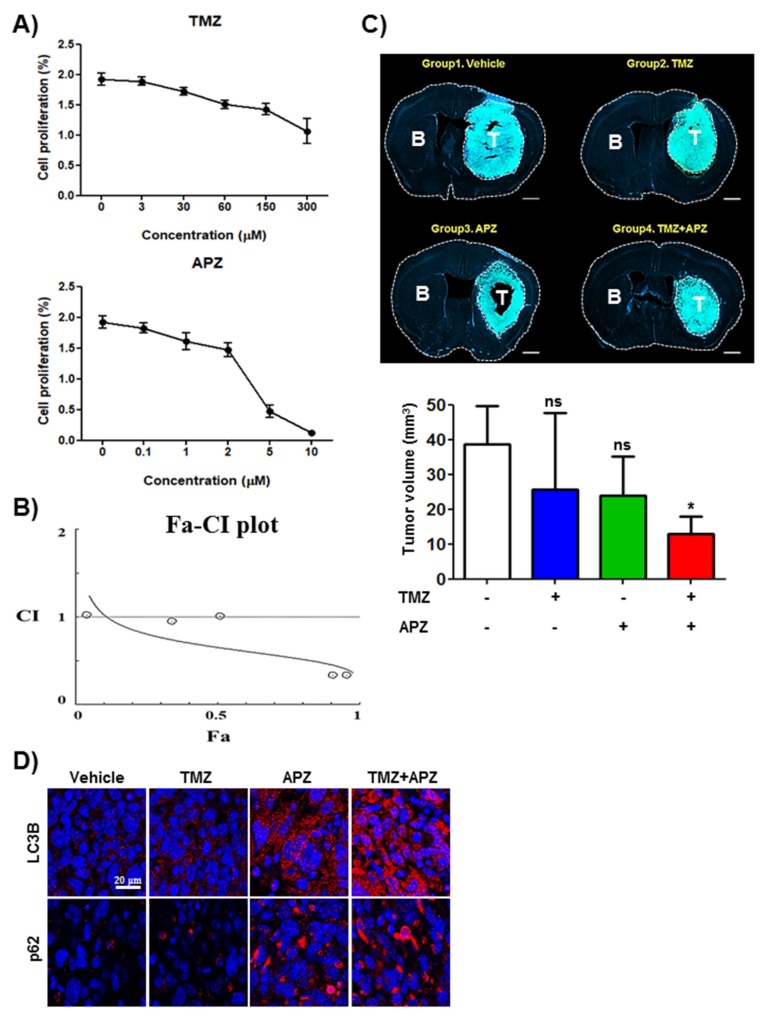
APZ shows the outstanding combinational effects with temozolomide (TMZ) in vitro and in vivo. (**A**) Effect on TMZ or APZ on the proliferation of GFP-GL261 in vitro. All cells were treated with TMZ or APZ for 48 h as indicated and cell growth was measured using an MTT calorimetric assay. *N* = 3 means ± SE. (**B**) Combination index plot for TMZ with APZ in GFP-GL261. Combination index (CI) plotted against fractions affected (Fa) analyzed using COMPUSYN software, which is a general software for dose and effect Pharmaco-Dynamics (PD) and Bio-Dynamics (BD). (**C**) Synergic effects of TMZ and APZ on tumor growth in vivo. Mice bearing glioma consisting of GFP-GL-261 cells in brain were treated with vehicle, TMZ (25 mg/kg), APZ (10 mg/kg), and combinational treatment of TMZ (25 mg/kg) with APZ (10 mg/kg) every other day (B: Brain region, T: Tumor region). Tumor volume of the mice in each group (*n* = 4) were measured over 18 days. *p < 0.05. (**D**) Representative images showed the immunofluorescence staining of p62 and LC3B (red) in brain glioma sections. Nuclei were stained with DAPI (blue). Scale bar, 20 μm.

Similar to effectiveness in U87MG, cell proliferation of GFP-GL261 cells was also inhibited by APZ treatment with IC_50_ values below 5 μM (Figure 3A) and exhibited significant synergism in combinational effect with TMZ in GFP-GL261 at 72 h (Figure 3B). Next, we evaluated tumor progression bearing orthotropic glioma derived from GFP-GL261 (Figure 3C). APZ alone treatment over the 18-days treatment had reduced implanted tumor volume such as TMZ alone treatment (vehicle-treated group: 39 mm^3^ [100%], TMZ-treated group: 26 mm^3^ [67%], APZ-treated group: 24 mm^3^ [62%], 18 days). Remarkably, cotreatment of TMZ with APZ exhibited dramatically combinational effectiveness (TMZ and APZ cotreated group: 13 mm^3^ [33%]). To analyze combinational effect of APZ with TMZ, CI value was calculated in an orthotropic glioma model. CI value for APZ in combinational effect with TMZ for antitumor effect in orthotropic glioma derived from GFP-GL261 was 0.49. Collectively, these results demonstrate that APZ exhibits significant synergism with TMZ both in vitro and in vivo. To investigate whether APZ inhibits autophagic flux in vivo as well, p62 and LC3B puncta were stained in these agents-treated group (Figure 3D). Immunofluorescent analysis demonstrated that both APZ alone treatment and combinational treatment of TMZ with APZ increased the accumulation of p62 and LC3-II in the brain glioma tissues compared with the vehicle or TMZ alone treated group. Additionally, combinational treatment of TMZ with APZ significantly recovered vessel abnormality by inhibiting vascular invasion from brain to glioblastoma region (Figure S4). These results demonstrate that APZ can be used as a potent therapeutic drug candidate to get over unassailable brain cancer.

### 2.3. DARTS and LC–MS/MS Analysis for Identification of APZ Protein Targets

DARTS is a label-free method for target identification of bioactive small molecules that exploits changes in protein proteolytic susceptibility [22]. To identify the protein targets of APZ, we developed a new method combining DARTS with liquid chromatography/tandem mass spectrometry (DARTS and LC–MS/MS) that improves the labor-intensiveness of traditional affinity-based methods. In this new combined method, the small molecule is exposed to the cell lysate, a proteome pool, to allow small molecule–protein target interactions. The stability of the target protein then increases, rendering it to be resistant to pronase treatment because of conformational changes induced by tight binding of the small molecule. Proteins with no or weak binding affinity with the small molecule are degraded, and proteins with high affinity binding resist protease treatment are identified by LC–MS/MS analysis.

**Figure 4:**
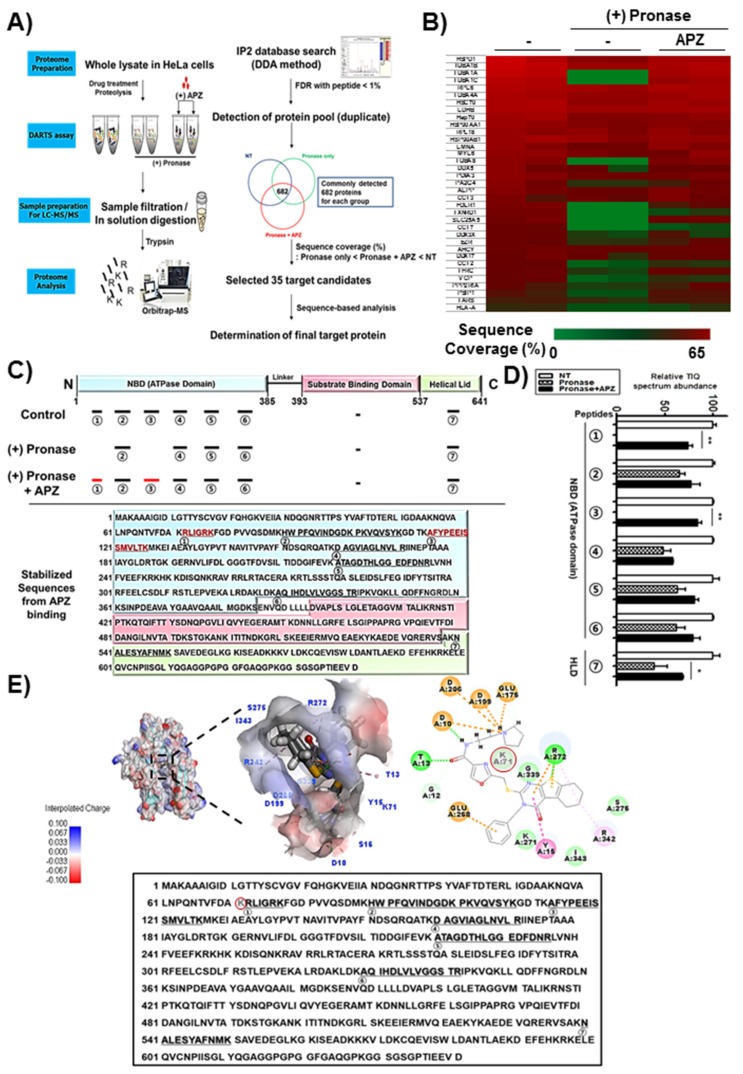
Combined drug affinity responsive target stability (DARTS) and LC−MS/MS method identified a new protein target of APZ. (**A**) Schematic diagram of the DARTS and LC–MS/MS method. A label-free DARTS and LC–MS/MS method was applied to identify the protein target of APZ. First, a DARTS assay with pronase digestion was applied for three groups: (1) Control, (2) pronase-alone treated, (3) pronase treated with APZ in duplicate. Second, LC–MS/MS analysis of the proteome protected by the small molecule followed by protein searching based on peptides using the IP2 database were conducted. Finally, 35 protein target candidates were selected with reasonable sequence coverage values (pronase alone < pronase treated with APZ < control). Filtering based on function by known phenotype narrowed down functional targets. Rescue of sequence coverage in specific domain suggests binding specificity and a possible binding site. Certain domains were protected. (**B**) Heatmap analysis of 35 candidate protein targets with reasonable sequence coverage values justifying the selection of Hsp70 as a potential target. Sequence coverage was calculated by dividing the number of amino acids observed by the whole amino acid length. (**C**) Sequence coverage identification Hsp70 peptides that bind APZ. The recovered sequences consisted of one in the NBD (nucleotide-binding domain; ATPase domain; ① RLIGRK, ③ AFYPEEISSMVLTK). (**D**) TIQ (top isotope quantification) peptides quantification [23]. Seven analyzed peptides and actual quantification of peptides. Values are mean ± SEM; *n* = 2. (**E**) In silico docking model of APZ interacting with Hsp70 (human Hsp70 NBD, RCSB PDB ID: 3ATV). In the left panel, APZ binds to the Hsp70 NBD pocket in the most stable pose and binding motifs are depicted with several high-affinity interactions between APZ and the Hsp70 NBD pocket. Ligands are shown as gray sticks in displayed charge receptor surfaces. Bonds are shown as dashed lines color-coded as follows: Hydrophobic interactions in orange, electrostatic interaction in purple, and hydrogen bonds in green and sky blue. In the right panel, the amino acids in whole Hsp70 sequences overlaid between stabilized peptide sequences and interacting with them directly with APZ in silico docking model (red circles with semi-transparent inner gray).

Our DARTS and LC–MS/MS method consists of five major steps and is summarized in the schematic diagram shown in Figure 4A: (1) Exposure of the small molecule to proteins in the cell lysate proteome pool, to allow for small molecule–protein target interactions, (2) pronase digestion of proteins unprotected by the small molecule, where proteins that bind resist protease treatment while proteins with little or no binding to the small molecule are degraded (Figure S5A), (3) further proteolysis with trypsin and LC–MS/MS analysis of resulting peptides to determine sequence coverage, (4) identification of proteins protected by the small molecule based on its enhanced sequence coverage by more than 3% compared to controls [23], and (5) selection of likely protein targets based on candidates with phenotypic relevancy to the small molecule [24,25].

Thirty-five candidate protein targets showed reasonable sequence coverage values that increased significantly upon APZ treatment compared to controls in the absence of APZ, as shown by heatmap analysis (Figure 4B, Table 1). High protein sequence coverage means higher confidence in interactors than proteins with low sequence coverage values. This enables interpretation of diverse transition patterns of sequence coverage with pronase. To select the protein target of APZ, sequence-based peptide analysis on DARTS and LC–MS/MS information was applied. As shown in Figure 1, we demonstrated that APZ inhibited autophagic flux. Inhibition of autophagic flux has been reported to be mainly caused by lysosomal stability, integrity, and functionality [26,27,28,29]. Among 35 candidate protein targets, Hsp70 modulates lysosomal integrity and chaperone function, and thus this protein was selected as a key binding protein of APZ and applied for sequence-based peptide analysis. Recovery of sequence coverage at specific locations reveals binding specificity and candidate binding sites between a small molecule and cognate protein target sequences. Sequence coverage analysis revealed that specific peptide fragments in the nucleotide binding domain (NBD, ATPase domain) of Hsp70 were protected by APZ in the pronase-treated proteome (Figure 4C). Since the NBD of Hsp70 is reportedly essential to its molecular function as a chaperone and protein interactions with cochaperones [30,31], we focused on this domain as a specific binding region of Hsp70 for APZ. Additionally, we analyzed seven peptides of pronase nontreated control sample detected in DARTS and LC–MS/MS proteome analysis as shown in Figure 4C,D. Seven peptides were stabilized by APZ treatment more than 10 % compared to pronase alone treatment (Figure S5B). Six of these peptides belong to NBD and one was a helical lid domain. Notably, two peptides of NBD (① and ③ of Figure 4D) were not detected by MS/MS in the absence of APZ after treatment with pronase. Interestingly, K71, G339, and R342 in stabilized peptides by APZ treatment were included in the NBD of Hsp70. However, ⑥ stabilized peptide by APZ treatment showed mild increase (17%) of its quantification without the significant difference compared to pronase alone treatment, suggesting that APZ may interact this peptide as a transient or weak binding. Therefore, K71 in ① stabilized peptide is most likely a tightly interacting amino acid residue of Hsp70 with APZ as shown in Figure 4E (red circle). This result also demonstrated that K71 plays significant roles for the direct binding of APZ to the NBD of Hsp70. In silico docking showed that APZ could directly bind to the NBD of Hsp70 (Figure 4E). Many charged ion groups and nonpolar rings of APZ can also contribute to its binding of the NBD in the same manner as ATP (Figure S5C) and VER-155008 (Figure S5D), a known Hsp70 inhibitor. In addition, these two ligands also shared a key binding amino acid of Hsp70, K71, in ① stabilized peptide of the NBD of Hsp70 by APZ treatment.

The chemical properties of APZ facilitate ionic charge interaction, noncovalent, and hydrogen bond formation via interactions between D10, K71, K271, and R272, which are common amino acids interacting with APZ, ATP, and VER-155008 in NBD, with high binding affinity (CDOCKER energy of ATP: −54.18 kcal/mol; VER-155008: −22.95 kcal/mol; APZ: −21.68 kcal/mol). Especially, charge interaction between amide group of APZ and aspartic acids (D10, D199, D206) of inner pocket in NBD of Hsp70 can play significant roles in the potent and high affinity binding of APZ to the NBD of Hsp70. These results also demonstrate that combined DARTS and LC–MS/MS-based target identification is an effective platform for identifying novel cellular protein targets of untagged small molecules.

### 2.4. Characterization of Hsp70 as a Potential Protein Target of APZ

**Figure 5:**
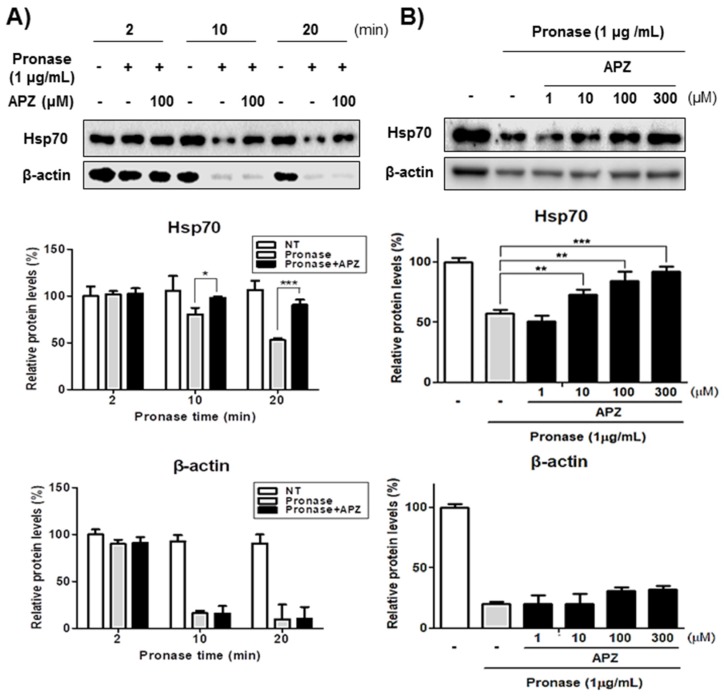
Characterization of Hsp70 as a potential target of APZ. (**A**) DARTS assay for target validation. Hsp70 and β-actin protein stability were unaltered upon APZ treatment in HeLa cell lysates. Pronase treatment was conducted for 2, 10, and 20 min. The graph plots the quantification data shown in b. *n* = 3, *p < 0.05, ***p < 0.001. (**B**) The DARTS assay demonstrated a dose-dependent binding of APZ to Hsp70. Treatment with 1 μg/mL pronase was conducted for 20 min. The graph plots the quantification data shown in b. *n* = 3, **p < 0.01, ***p < 0.001. More details of western blot, please view at the supplementary materials.

The DARTS assay with Western analysis alone could be also used to validate the LC–MS/MS results by demonstrating the increased stability of Hsp70 after APZ treatment even after treatment with pronase for up to 20 min, whereas β-actin, a protein with no APZ binding affinity, was significantly digested by pronase (Figure 5A). Notably, the stability of Hsp70 was increased in a dose-dependent manner by APZ treatment, suggesting that APZ tightly interacts with Hsp70 (Figure 5B). Despite evidence suggesting that Hsp70 stabilizes lysosomal integrity by binding to endolysosomal anionic phospholipids [32], the mechanism by which Hsp70 affects autophagy has not been demonstrated so far. Our results indicated that APZ inhibits Hsp70 interaction or function on lysosomal integrity in autophagosomes by binding Hsp70.

### 2.5. Validation of Hsp70 as a Protein Target of APZ

**Figure 6:**
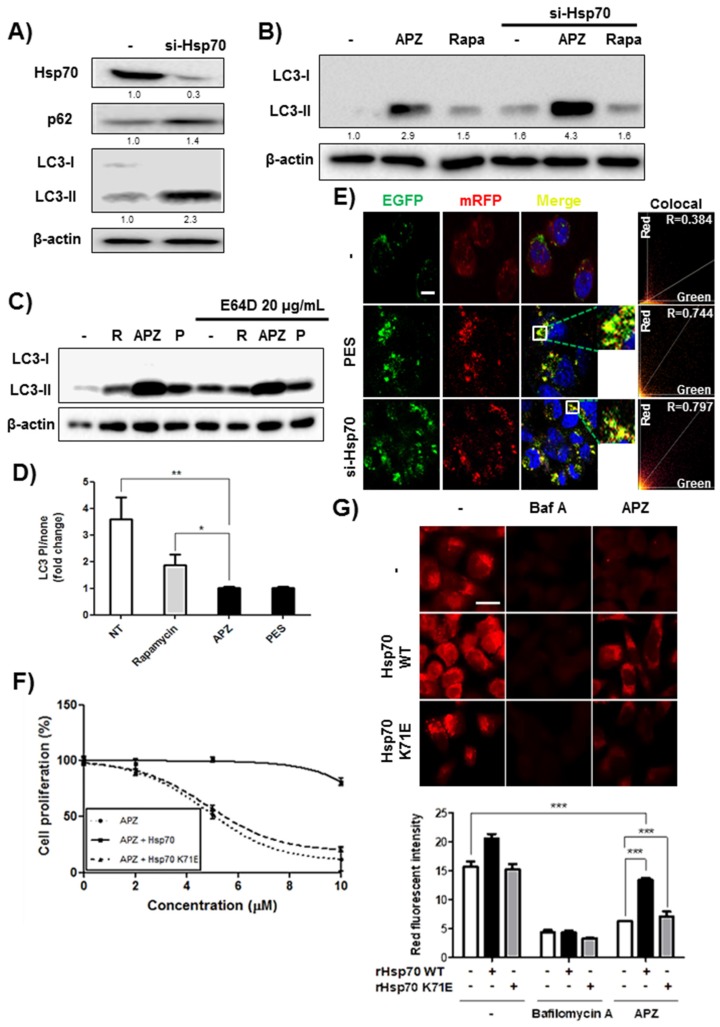
Validation of Hsp70 as a protein target of APZ. (**A**) Immunoblots of p62, and LC3 in HeLa cells after 24 h of Hsp70 knockdown (10 nM). (**B**) Immunoblot of LC3 upon compounds treatment after Hsp70 knockdown (10 nM). Autophagy induction by APZ significantly increased after Hsp70 knockdown, but rapamycin did not have this effect. (**C**) LC3 conversion increased after E64D treatment for 24 h. E64D pretreatment did not affect APZ-induced LC3 conversion. (**D**) Quantification of data shown in b. Ratio of LC3-II in E64D-treated cells to that in untreated cells. Values represent means ± SEM of fold-changes in cotreatment relative to levels in untreated cells; *n* = 3. * *p* < 0.05, **p < 0.01, *p < 0.05. (**E**) Autophagic flux in HeLa cells as measured by mCherry-GFP LC3 after si-Hsp70 and 2-phenylethynesulfonamide (PES, 5 μM) treatment. Representative merged-channel images; scale bar, 10 μm. Pearson coefficient for the colocalization analysis is shown. (**F**) Genetic Hsp70 overexpression reduced antiproliferative activity of APZ. Values represent means ± SE; *n* = 4. Wildtype EGFP-Hsp70 or K71E EGFP-Hsp70 vectors (200 nM) were transfected into HeLa cells for 4 h. APZ was administered at the indicated concentrations for 48 h. (**G**) Acridine orange staining was conducted to visualize lysosomal integrity. After transfection of wildtype EGFP-Hsp70 or K71E EGFP-Hsp70 vectors (200 nM) into HeLa cells, the samples were treated with APZ (5 μM) and bafilomycin A (10 nM) for 6 and 24 h. Cells were treated with acridine orange (2 μg/mL) for 20 min before fixation. After fixation, each sample was imaged by confocal microscopy. Scale bar, 20 μm. Graph of fluorescence in g. Values represent means ± SEM; *n* = 30 cells. ***p < 0.001. More details of western blot, please view at the supplementary materials.

To validate the biological relevance of Hsp70 on autophagy, we explored the effects of Hsp70 knockdown on autophagy. As shown by immunoblotting for LC3-II, a component of the autophagosome membrane, and other autophagic marker, p62 [33], Hsp70 knockdown mimicked the same activities at the p62 and LC3-II levels as did APZ treatment (Figure 6A, Figure S1). Furthermore, APZ treatment of Hsp70-knockdown cells resulted in higher LC3-II expression than that observed following APZ treatment alone (Figure 6B). Thus, APZ’s mode of action involves Hsp70 inhibition, as LC3-II induction by APZ increased greatly upon Hsp70 knockdown. In contrast, rapamycin-induced autophagy was unaffected by Hsp70 knockdown (Figure 6B).

PES, a known Hsp70 inhibitor that also binds its substrate-binding domain (SBD) and induces lysosomal dysfunction [34,35] exhibited similar biological activities as APZ on LC3-II levels with and without E64D (Figure 6C,D). Furthermore, PES and Hsp70 knockdown inhibited autophagy flux, similar to APZ treatment (Figure 6E). Hsp70 was overexpressed using an EGFP-Hsp70 clone using wildtype (WT) Hsp70 and a form with a mutation in the ATP-binding motif, Hsp70 K71E [36] (Figure S6B**)**. Overexpression of WT Hsp70 rescued the cell growth inhibition caused by APZ both in cancer (HeLa) and normal cell (Chang) lines, but overexpression of mutant-type (MT) Hsp70 did not (Figure 6F, Figure S6C). Moreover, the lysosome inhibitory activity caused by APZ treatment was rescued by overexpressing WT Hsp70, but not by overexpressing MT Hsp70. Notably, neither WT nor MT Hsp70 rescued lysosome inhibition caused by bafilomycin A treatment, a well-known V-ATPase inhibitor of lysosomes [37] (Figure 6G). These results clearly demonstrate that Hsp70 is a biologically relevant protein target of APZ responsible for its autophagy-inhibiting mechanism.

To further investigate whether APZ shows on-target effects in normal and cancer cell lines, the expression level of Hsp70 was examined in four cell lines (Chang, HEK293: Normal cell lines; HeLa, U87MG: Cancer cell lines) to establish a correlation between APZ and its potential target.

**Figure 7:**
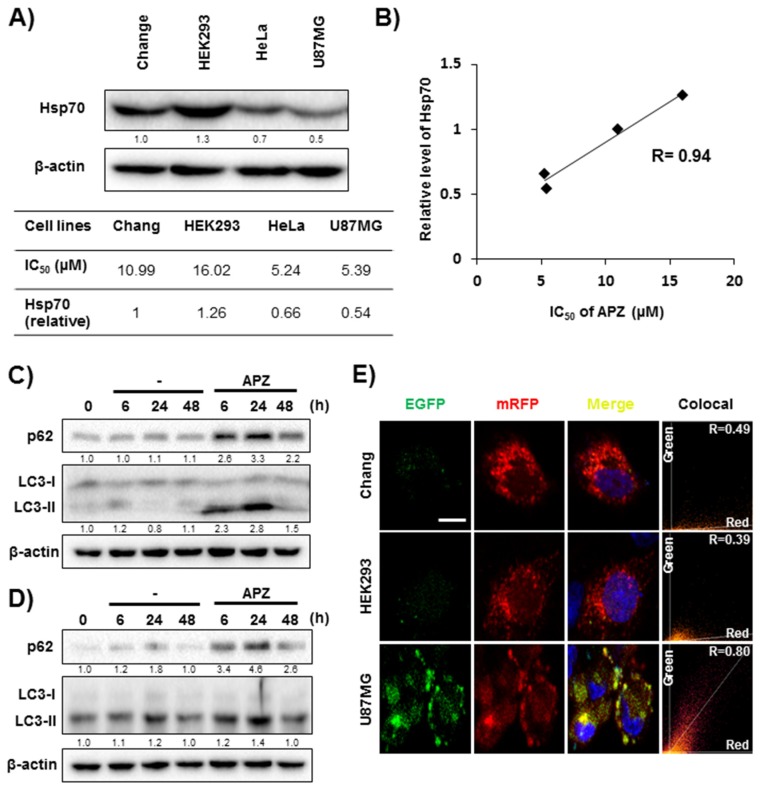
Efficacy of APZ is dependent on the expression level of Hsp70. (**A**) Expression levels of Hsp70 and IC_50_ values from APZ treatment for 48 h both in normal cell lines (Chang, HEK293) and cancer cell lines (HeLa, U87MG). (**B**) Pearson correlation graph between IC_50_ of APZ and expression level of Hsp70 in various cell lines. (**C**–**D**) LC3-II and p62 levels after compound treatment for 6, 24, and 48 h. APZ treatment (5 μM) induced autophagic degradation both LC3-II and p62 levels from 24–48 h in Chang (**C**) and HEK293 (**D**) cell lines. (**E**) Autophagic flux evaluation in various cells using mCherry-GFP LC3 in the presence of APZ (5 μM, 24 h). Representative images of merged channels are shown; scale bar, 10 μm. Pearson coefficient for the colocalization analysis is shown. More details of western blot, please view at the supplementary materials.

Protein Hsp70 (Figure 7A). As a result, the expression level of Hsp70 in normal cells was higher than that of cancer cells, which is consistent with results in Figure 6. Then, IC_50_ values, which was monitored in cell proliferation assay with four cell lines (Figure 2D), were calculated. Pearson correlation coefficient was used to validate the relationship between Hsp70 expression and APZ efficacy on cell proliferation. Notably, there is a significantly positive correlation (*r* = 0.94) between the expression level of Hsp70 and the IC_50_ of APZ (Figure 7B). In addition, the effects of APZ on autophagy degrading markers, p62 and LC3-II kinetics, in normal cell lines were examined to assess autophagic flux. APZ treatment rapidly increased the levels of p62 and LC3-II in 24 h and then decreased at 48 h both in Chang (Figure 7C) and HEK293 (Figure 7D) cell lines, indicating the degradation of the proteins at the late stage of autophagy. For further validation, a double-tagged LC3 (mRFP-GFP) plasmid was transfected in Chang, HEK293, and U87MG cell lines. Notably, APZ treatment resulted in increase in red fluorescence (autolysosomes) in Chang and HEK293 cells indicating autophagy flux, whereas resulted in increase in yellow fluorescence (autophagosomes) in U87MG cells (Figure 7E) indicative of autophagy inhibition. These results demonstrated that the efficacy of APZ depends on its target protein, Hsp70.

### 2.6. APZ Inhibits Lysosomal Integrity by Antagonizing Hsp70

**Figure 8:**
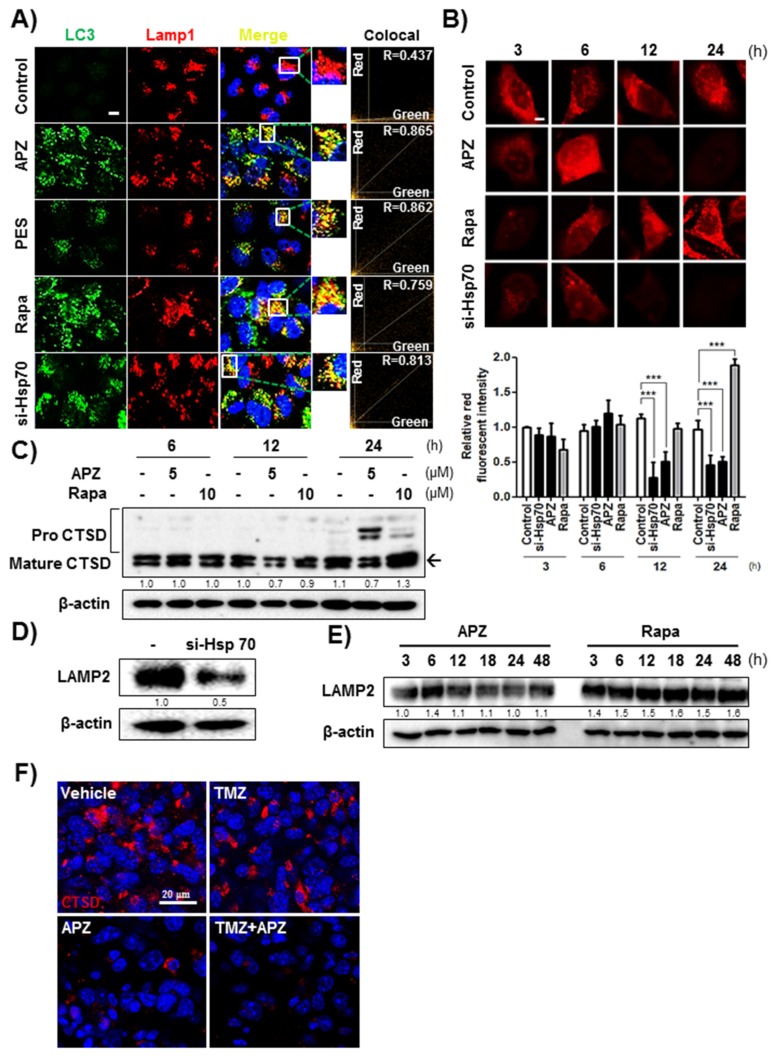
Hsp70 inhibition by APZ affects lysosomal integrity. (**A**) Immunocytochemistry staining with red fluorescent protein (RFP)-tagged LAMP1 and green fluorescent protein (GFP)-tagged LC3 with Hsp70 siRNA transfection or compounds in HeLa cells. The samples were treated with APZ (5 μM), PES (5 μM), rapamycin (10 μM), and si-Hsp70 vector (10 nM) for 24 h. The colocalization of GFP-LC3 and RFP-LAMP1 was monitored by confocal microscopy. Scale bar, 20 μm. (**B**) Acridine orange staining was used to visualize lysosomal integrity. The samples were treated with APZ (5 μM), rapamycin (10 μM), and si-Hsp70 vector (10 nM) for 24 h. Cells were treated with acridine orange (2 μg/mL) for 20 min before fixation. After fixation, samples were examined by confocal microscopy. Scale bar, 10 μm. Graph indicates fluorescence quantification. Values are means ± SEM; *n* > 30 cells. ***p < 0.001. (**C**) Immunoblot analysis indicating altered processing of cathepsin D from the prevalent premature form to the mature form after APZ treatment (5 μM) for 24 h. (**D**) Immunoblot analysis upon si-Hsp70 transfection (10 nM) indicating decreased expression of LAMP2 in HeLa cells. (**E**) Expression of LAMP2 decreased alone upon treatment with APZ (5 μM) for 48 h. (**F**) Representative images showed the immunofluorescence staining of cathepsin D (CTSD) (red) in brain glioma sections. Nuclei were stained with DAPI (blue). scale bar, 20 μm. More details of western blot, please view at the supplementary materials.

An accumulation in cellular cargo proteins can be caused at any step after autophagosome formation and can be induced either by inhibiting fusion of autophagosomes with lysosomes or losing the ability for lysosomal proteolysis. Therefore, we explored whether impaired autophagy induced by APZ and Hsp70 inhibition might be caused by interrupting fusion between both compartments. Examination of the LC3-marking autophagosomes and LAMP1-labeled lysosomes in response to Hsp70 inhibition including Hsp70 knockdown and inhibitors (APZ, PES) suggested that Hsp70 inhibition did not decrease the colocalization of LC3 and LAMP1-labeled lysosomes similar to rapamycin known as a fusion inducer (Figure 8A). Thus, Hsp70 inhibition does not compromise or delay the fusion of autophagosomes with lysosomes. An impaired autophagosomal clearance after APZ treatment led us to assess whether lysosomal integrity was affected by APZ. APZ treatment reduced lysosomal integrity for 12–24 h. Moreover, Hsp70-deficient cells exhibited the same reduction in lysosomal integrity from 6 h, whereas rapamycin treatment significantly increased acridine orange intensity, indicating that rapamycin induces autophagic flux by activating lysosome activity (Figure 8B). Thus, both inhibition of Hsp70 by APZ and its genetic knockdown inhibit lysosomal integrity.

To investigate the effect of APZ on lysosomal integrity and enzyme activity, the lysosomal enzyme cathepsin D [38] was examined in HeLa cells after treatment with APZ and rapamycin (Figure 8C). APZ treatment reduced the levels of the mature form of cathepsin D in a time-dependent manner, whereas rapamycin did not; rather, it induced the mature form of cathepsin D as demonstrated by acridine orange staining of lysosomes (Figure 8B). Thus, APZ inhibits lysosomal integrity by antagonizing Hsp70. LAMP2 protein levels reflect the size and number of lysosomal compartments [39], providing a correlation between lysosomal integrity and APZ. Upon treatment with APZ and Hsp70 knockdown, LAMP2 levels significantly decreased compared to those in untreated or rapamycin-treated cells (Figure 8D,E), demonstrating that APZ inhibits lysosomal integrity by binding Hsp70. Additionally, cathepsin D (CTSD) with four agents-treated brain glioma sections (Figure 3C,D), were stained to validate whether APZ inhibits lysosomal integrity leading to autophagic cell death in the orthotropic xenograft model (Figure 8F). Staining of tumor tissues for CTSD showed a diffuse pattern in the vehicle- or TMZ alone-treated group, whereas the significantly reduced levels were observed in APZ alone- or cotreatment of APZ with TMZ group. These results suggest that Hsp70 regulates the activities affected by APZ, such as lysosomal formation, autophagic flux, and autophagic cell death, and that the NBD of Hsp70 is required for this regulation.

## 3. Discussion

Autophagy inhibitors that induce autophagic cell death have great potential as valuable new anticancer therapeutics [40], but the precise autophagy mechanism has been established for surprisingly few anticancer drugs. As a recent case, compound CA-5f was identified as a novel late stage autophagy inhibitor by interrupting fusion between autophagosomes and lysosomes [41]. CA-5f exhibited selective cytotoxicity against A549 nonsmall cell lung cancer (NSCLC), which is a resistant cancer cell to radiation or chemotherapy, than normal human umbilical vein endothelial cells (HUVECs) by inhibiting autophagic flux without affecting lysosomal functionality. This study presented that discovering novel autophagy inhibitors could be one of the promising strategies for clinical application and recent advances in cancer. Although few autophagy inhibitors targeting cancer have been developed, discovering new autophagy inhibitors can reveal new autophagy mechanisms and so provide new routes for the development of new cancer-targeting agents.

The unique features of APZ as a new Hsp70 inhibitor come from its physicochemical structure and properties distinct from those of known Hsp70 inhibitors leading to high hydrophobic property (LogP: 4.9) and tight binding to nucleotide binding domain (NBD) of Hsp70. APZ binds with Hsp70 through hydrophobic interaction mainly, which is pivotal for formation of rigid complex and tight binding [42]. For example, VER-155008 has been reported to competitively bind to ATP in NBD and inhibits ATPase and chaperon activity by binding tightly with NBD leading to cell death [43]. In these studies, APZ induced apoptosis-independent and cancer specific cell death without affecting the growth of normal cell lines or mice and the effects of both compounds were rescued by genetic overexpression of wildtype Hsp70. Notably, APZ exhibited significant synergism with temozolomide, a chemotherapeutic drug of glioblastoma, in vitro and orthotropic mouse model. Treatment of malignant glioblastoma has remained a challenge because it requires complete elimination of the residual tumor, which leads to fatal recurrence, and blood-brain barrier (BBB) blocks delivery of anticancer drugs. Therefore, potent drugs complementing these limitations must be developed. APZ shows the remarkable anticancer effects through the significant synergism with the existing drug of TMZ both in subcutaneous and orthotopic tumor mouse models.

Our in silico docking studies of three ligands including ATP, APZ, and VER-155008 induced a strong interaction with NBD through a similar binding motif. The tight binding property of APZ with Hsp70 can contribute its cancer cell specific cell death activity and provide a tool compound to explore Hsp70 as a new molecular probe for Hsp70 distinct from currently known Hsp70 inhibitors such as PES, VER-155008, and apoptozole [44]. We propose that APZ is a new molecular probe that selectively targets cancer as a new Hsp70 inhibitor and is a unique tool for basic studies of autophagy and cell death mechanisms. Collectively, APZ can be used as a new molecular probe to elucidate new roles of Hsp70 with its unknown functions including autophagosomes formation, and lysosome blocker with autophagosomes that might provide the new biological and mechanistic insights on Hsp70 in future studies.

We found that regulation of Hsp70 expression by both transient knockdown and overexpression specifically affected the biological activities of APZ. These results are consistent with VER-155008, which has been reported to rescue the inhibition of cell proliferation when Hsp70 was transiently overexpressed whereas the biological effects was potentiated by cotreatment with Hsp90 inhibitor [45]. Thus, our results highlight Hsp70 as a promising therapeutic target for cancer treatment. Selective inhibition of cell proliferation in cancer cells over normal cells and the ability of APZ to suppress xenograft tumors suggest the potential of APZ as an anticancer therapeutic. In agreement with our proposed model, treatment of APZ to a glioblastoma mouse xenograft model resulted in significant suppression of lysosome function.

## 4. Materials and Methods

### 4.1. Compounds and Antibodies

APZ was screened from among 2000 chemicals obtained from the structure-based synthetic and natural product libraries of ChemDiv, Chemgenex, and Korea Chemical Bank. All compounds were stored as stock solutions in 10 mM DMSO at −20 °C and diluted with Dulbecco’s Modified Eagle Medium (DMEM) before in vitro experiments. The working solution was freshly prepared in a basal medium, and the control group was treated with the same amount of DMSO as a vehicle control. Bafilomycin A1, rapamycin, dithiothreitol (DTT), trifluoperazine, iodoacetamide (IAA), formic acid, E64D cysteine protease inhibitor, pifithrin-μ (PES), and artesunate (ART) were purchased from Sigma-Aldrich (St. Louis, MO, USA). Pronase and protease inhibitor cocktail tablets were obtained from Roche (Basel, Switzerland). Phosphatase inhibitor solution and Triton X-100 were purchased from Sigma-Aldrich. Hoechst 33342, Lipofectamine LTX, Lipofectamine 2000, DMEM, RPMI, MEM, and fetal bovine serum (FBS) were purchased from Invitrogen (Carlsbad, CA, USA). Antilight chain 3 (LC3) antibody was purchased from MBL (Nagoya, Japan). Anti-ATG16L1 antibodies was purchased from Cell Signaling Technology (Danvers, MA, USA), and antiHsp70, anticathepsin D (CTSD), antiLAMP2, antiPARP, and antiβ-actin antibodies were purchased from Abcam (Cambridge, UK). Coomassie blue and silver staining dye were purchased Thermo Fisher Scientific (Waltham, MA, USA). mRFP-GFP-LC3B plasmids were gifts from Dr. Jaewhan Song at Yonsei University (Seoul, Korea).

### 4.2. Cell Culture

HeLa (human cervical cancer), A549 (human lung carcinoma), HEK293 (human embryonic kidney), Chang (human liver), and EGFP-LC3 stably expressing COS7 (monkey kidney) cells were grown in DMEM containing 10% FBS and 1% antibiotics. U87MG (human glioblastoma) cells were grown in a minimum essential medium eagle (MEM) containing 10% FBS and 1% antibiotics. All cell cultures were maintained at pH 7.4 in a humidified incubator at 37 °C under 5% CO_2_ in air.

### 4.3. Hsp70 RNA Interference by Reverse Transcription Polymerase Chain Reaction and Recombinant Hsp70 Plasmid Transfection Analysis

Human Hsp70-specific small interfering RNA (siATP6V1A) was from GE Healthcare Dharmacon (Lafayette, CO, USA). The ON-TARGETplus/SMARTpool-derived siRNA against human Hsp70 mRNA was named as L-005168-00-0005. For knockdown of Hsp70 mRNA, HeLa cells were transfected with random or Hsp70 siRNA using Lipofectamine 2000 transfection reagent (Invitrogen) according to the manufacturer’s instructions. The presence of Hsp70 mRNA was validated by quantitative reverse transcription polymerase chain reaction (qRT-PCR) analysis using primers specific for the Hsp70 NBD (sense, 5′-GAA TTC ATG GCC AAA GCC GCG-3′; antisense, 5′-CTC GAG CTC GGA CTT GTC CC-3′). GAPDH mRNA was used as a reference for qRT-PCR. For transient overexpression of Hsp70, both DNA vectors, including pEGFP Hsp70 (Lois Greene Lab plasmid # 15215; Addgene, Cambridge, MA, USA) and pEGFP Hsp70 K71E (Lois Greene Lab plasmid # 15216; Addgene) were purchased. For overexpression of Hsp70, HeLa cells were transfected with negative control or Hsp70 pEGFP Hsp70 or pEGFP Hsp70 K71E using Lipofectamine LTX transfection reagent (Invitrogen) according to the manufacturer’s instructions. The presence of Hsp70 protein was validated by immunoblotting analysis.

### 4.4. In Silico Docking Study

All molecular docking analyses were performed with Discovery Studio 2016 software (Accelrys, San Diego, CA, USA) adopting the CHARMm force field. The crystal structure of the human Hsp70 nucleotide-binding domain (PDB ID 3ATV) was obtained from the RCSB protein data bank. The protein structures of the Hsp70 nucleotide-binding domain were energy-minimized using the Powell algorithm. The ligands were docked using Ligandfit. The Ligandfit parameters were validated using the ligand from the Hsp70 nucleotide-binding domain crystal structure. APZ was docked to the ATPase domain of the protein, and nine poses were generated. The most predictive binding modes were determined based on various scoring functions (Ligscore1_Dreiding, Ligscore2_Dreiding, PLP1, PLP2, PMF, DOCK_SCORE), and binding energies were calculated in Ligandfit.

### 4.5. Immunoblotting

Soluble proteins were harvested from cells by using a SDS lysis buffer (50 mM Tris HCl at pH 6.8 containing 10% glycerol, 2% SDS, 10 mM dithiothreitol, and 0.005% bromophenol blue). Equal volumes of proteins were separated by 8% or 12.5% SDS-PAGE and transferred to polyvinylidene fluoride membranes (EMD Millipore, Billerica, MA, USA). Blots were then blocked and immunolabeled overnight at 4 °C with primary antibodies, antiLC3 (MBL), PARP, p-mTOR, mTOR, p-S6K, S6K, Atg16L1 (Cell Signaling Technology), p62 (BD Biosciences, Franklin Lakes, NJ, USA), GFP, Hsp70, Cathepsin D, LAMP2, and actin (Abcam). Immunolabeling was visualized using an enhanced chemiluminescence kit (Amersham Life Science, Inc., Amersham, UK) according to the manufacturer’s instructions. Images were quantified using Image Lab (Bio-Rad, Hercules, CA, USA). Actin was used as an internal control.

### 4.6. Immunocytochemistry Staining

To determine the effect on the fusion between autophagosomes and lysosomes, HeLa cells were seeded at a density of 1.5 × 10^5^ cells/well in 6-well plates and incubated overnight. Following incubation overnight, the cells samples were fixed with 4% paraformaldehyde and washed with PBS three times. Then, the cells were treated with compounds or si-Hsp70 for the time periods indicated, followed by treatment with LC3 and LAMP1 primary antibodies (Abcam, Cambridge, UK) for 1 h. Nuclei were stained with DAPI. Images were obtained using a confocal microscope at a 400× magnification.

### 4.7. ATP-Monitoring Luminescence Assay

To determine the effect of cellular ATP levels on cell proliferation, an ATPlite 1-step Luminescence Assay Kit (Perkin Elmer, Waltham, MA, USA) was purchased and used according to the manufacturer’s instruction. HeLa cells were seeded in 96-well white plates at 2000 cells/well and incubated overnight. After drug treatments, luminescence was measured using a Victor 3 Multilabel Plate Reader (Perkin Elmer).

### 4.8. Cell Proliferation Assay

All cells were seeded in 96-well plates at 3000 cells/well and incubated overnight. Drugs were added to the cells to determine their effects on cell proliferation. Cells were grown for 48 h, and growth was analyzed by the 3-(4,5-dimehylthiazol-2-yl)-2,5-diphenyl tetrazolium bromide (MTT) (Sigma-Aldrich) colorimetric assay. MTT-formazan in each well was dissolved in 150 μL DMSO, and the absorbance at 540 nm was read with a microplate reader.

### 4.9. Cell Death Assay

HeLa cells were seeded at 1 × 10^4^ cells/well in a 12-well plate and incubated overnight at 37 °C. One hour before treatment with APZ, inhibitors of cell death including Z-VAD-FMK, necrostatin-1, and liproxstatin-1 were added. After 24 h, cell viability was determined by trypan blue staining.

### 4.10. Transmission Electron Microscopy

Cells were harvested by trypsinization, washed twice with phosphate-buffered saline (PBS), and fixed with 2% paraformaldehyde/2% glutaraldehyde in a 0.1 M phosphate buffer at pH 7.4. After fixation, the cells were washed with sodium cacodylate, scraped from the plates, and pelleted by centrifugation. Cell pellets were post-fixed with 1% (*v/v*) osmic acid in sodium cacodylate and stained with 1% uranyl acetate. After dehydration, the pellets were embedded in Durcopan (Sigma-Aldrich). Ultrathin sections were prepared using an LKB 8800 Ultratome III and observed with a Philips EM 400T transmission electron microscope.

### 4.11. DARTS Assay

Cell lysates were obtained from HeLa cells. Cells were scraped and lysed with a M-PER lysis buffer. After centrifugation for 15 min at 10,000× *g*, the supernatant was obtained, and protein content was quantified using the Bradford reagent. Before drug treatment, protein concentration was diluted to 1 mg/mL. Samples were treated with the drugs APZ and DMSO for 4 h at 4 °C. Samples were then incubated with pronase or distilled water, as indicated, for 2, 10, and 20 min at 25 °C. A small portion of each sample was used for Western blot analysis, and the remainder of each sample was frozen for use in the LC−MS/MS analysis. Actin was used as an internal control.

### 4.12. In Vivo Mouse Tumor Xenograft Assay

Mice were housed in a pathogen-free facility at the Laboratory Animal Research Center of Yonsei University (Seoul, Korea). These mice were handled in accordance with the Institutional Animal Care and Use Committee (permission number: IACUC-201511-527-01) and International Guidelines for the Ethical Use of Animals. For intracranial tumor models, 2 × 10^5^ GL261-GFP were implanted into the right hemispheres of 8–10-wk-old male experimental mice via stereotaxic injection. After 1 week, mice were randomly selected and separated into four groups (four mice per group) and intraperitoneally treated with vehicle or TMZ (25 mg/kg) or APZ (10 mg/kg) or combinational treatment (TMZ 25 mg/kg, APZ 10 mg/kg) every two days for 18 days. Tumor volume was calculated according to the formula 0.5 × A × B2, where A is the longest diameter of a tumor, and B is its perpendicular diameter. For subcutaneous tumor models, U87 glioblastoma cells (5 × 10^6^ cells) suspended in 200 μL PBS/Matrigel (1:1) were subcutaneously implanted into the dorsal flank of athymic nude mice (four-week-old female BALB/c nu/nu; Orient Bio, Seoul, Korea). Once the tumors became palpable (50–100 mm^3^, 10 days), mice were randomly selected and separated into two groups (five mice per group) and intraperitoneally treated with vehicle or APZ (10 mg/kg) every two days. Vehicle and drugs were dissolved in a saline:Ethanol:Tween 80 solution at a ratio of 88:2:18. The tumor volume and mouse body weight were measured daily using the following formula: π/6 × length × width × height. After 1 h of drug treatment, mice were sacrificed, and tissue samples were obtained. The tumors were surgically removed and slowly frozen with dry ice (−70 °C).

### 4.13. Acridine Orange Staining

HeLa cells were seeded at a density of 1.5 × 10^5^ cells/well in 6-well plates and incubated overnight. The cells were treated with drugs for the time periods indicated, followed by treatment with 2 μg/mL acridine orange (Sigma-Aldrich). For tissue staining, sequential frozen sections were cut from each tumor. Vehicle- and APZ-treated sections were stained with 5 μg/mL acridine orange. Nuclei were stained with DAPI. Following incubation for 20 min, the cells and tissue samples were fixed with 4% paraformaldehyde and washed with PBS three times. Images were obtained using a confocal microscope at a 400× magnification. Red fluorescence intensity was quantified using Image J (NIH, Bethesda, MD, USA).

### 4.14. mRFP-GFP-LC3B Plasmid Transfection

All cells were seeded in 6-well plates at 1.5 × 10^5^ cells/well and incubated overnight. All cells were transfected with negative control or mRFP-GFP-LC3B plasmids using Lipofectamine LTX transfection reagent (Invitrogen) for 4 h. The cells were treated with drugs for the time periods indicated, followed by treatment with mRFP-GFP-LC3B plasmid (1000 ng). Nuclei were stained with DAPI. Following incubation for 20 min, the cells and tissue samples were fixed with 4% paraformaldehyde and washed with PBS three times. Images were obtained using a confocal microscope at a 400× magnification.

### 4.15. Statistical Analysis

All data are expressed as the means ± SEM with GraphPad Prism (ver. 5.00 for Windows; GraphPad Software, Inc., San Diego, CA, USA). Data were obtained from at least three independent experiments. Statistical analyses were performed using an unpaired, two-tailed Student’s *t*-test. *P*-values less than 0.05 were considered statistically significant (* indicates *p* < 0.05, ** indicates *p* < 0.01, *** indicates *p* < 0.001).

## 5. Conclusions

Here, our findings demonstrated that the DARTS and LC–MS/MS method is a powerful strategy for the discovery of novel targets with the significant advantage over traditional affinity based approaches in that unmodified compounds can be profiled, so considerably speeding up discovery. The present study revealed how to interpret combined analysis results to filter perturbations that were likely relevant to the observed phenotype of APZ. We found that filtering based on function to a known phenotype of small molecule is a useful method for narrowing down functional targets. Importantly, the DARTS and LC–MS/MS method effectively circumvents the chemical modification and coupling to solid supports required by conventional strategies and is a simple and accessible method.

Collectively, our study showed that both APZ treatment and orthogonal Hsp70 inhibition increased specific markers of autophagy to form autophagosomes. In addition to the role of APZ as a specific inhibitor of Hsp70, we also revealed a new functional role of Hsp70 in autophagosome formation and induction of incomplete lysosomes leading to autophagic cell death in cancer cells. Moreover, we established the exact mechanisms of the functional role of Hsp70 to provide a further insight that can target cancer effectively. This novel role of Hsp70 likely involves the stabilization of lysosomes based on pH integrity and suggests a mechanism for its action.

## Figures and Tables

**Table 1:** Selected 35 proteins resistant to pronase with APZ treatment by LC–MS/MS analysis.

Gene Locus (Human)	Name	Full Name
sp|P10809|CH60	HSPD1	60 kDa heat shock protein, mitochondrial precursor
sp|P05141|ADT2	SLC25A5	ADP/ATP translocase 2
sp|Q99832|TCPH	CCT7	T-complex protein 1 subunit eta
sp|P15311|EZRI	EZR	Ezrin
sp|P15328|FOLR1	FOLR1	Folate receptor alpha
sp|P08107|HSP71	HSPA1A	Heat shock 70 kDa protein 1A/1B
sp|P11142|HSP7C	HSPA8	Heat shock cognate 71 kDa protein
sp|P07900|HS90A	HSP90AA1	Heat shock protein HSP 90-alpha
sp|P08238|HS90B	HSP90AB1	Heat shock protein HSP 90-beta
tr|B7Z904|B7Z904	TXNRD1	Thioredoxin reductase 1
sp|P07195|LDHB	LDHB	L-lactate dehydrogenase B chain
sp|P60660|MYL6	MYL6	Smooth muscle of Myosin light polypeptide 6
sp|O75475|PSIP1	PSIP1	PC4 and SFRS1-interacting protein
sp|P02545|LMNA	LMNA	Prelamin-A/C
sp|Q92841|DDX17	DDX17	Probable ATP-dependent RNA helicase DDX17
sp|P17844|DDX5	DDX5	Probable ATP-dependent RNA helicase DDX5
sp|Q9UQ80|PA2G4	PA2G4	Proliferation-associated protein 2G4
sp|P30101|PDIA3	PDIA3	Protein disulfide-isomerase A3
sp|P30153|2AAA	PPP2R1A	Serine/threonine-protein phosphatase 2A 65 kDa regulatory subunit A alpha
sp|P26639|SYTC	TARS	Threonyl-tRNA synthetase
sp|P02786|TFR1	TFRC	Transferrin receptor protein 1
sp|Q71U36|TBA1A	TUBA1A	Tubulin alpha-1A chain
sp|P68363|TBA1B	TUBA1B	Tubulin alpha-1B chain
sp|Q9BQE3|TBA1C	TUBA1C	Tubulin alpha-1C chain
sp|P68366|TBA4A	TUBA4A	Tubulin alpha-4A chain
sp|Q9NY65|TBA8	TUBA8	Tubulin alpha-8 chain
sp|P78371|TCPB	CCT2	T-complex protein 1 subunit beta
sp|O00571|DDX3X	DDX3X	ATP-dependent RNA helicase DDX3X
sp|Q02543|RL18A	RPL18	60S ribosomal protein L18
sp|Q02878|RL6	RPL6	60S ribosomal protein L6
sp|P01891|1A68	HLA-A	HLA class I histocompatibility antigen, A-68 alpha chain
sp|P49368|TCPG	CCT3	T-complex protein 1 subunit gamma
sp|P05187|PPB1	ALPP	Alkaline phosphatase, placental type
sp|P23526|SAHH	AHCY	Adenosylhomocysteinase
sp|P55072|TERA	VCP	Transitional endoplasmic reticulum ATPase

## Supplementary Materials

The following are available online at https://www.mdpi.com/2072-6694/12/3/543/s1, Figure S1: APZ may not induce autophagy continuously, but rather trigger incomplete autophagy resulting from autophagic flux inhibition, Figure S2: Effects of APZ on U87MG tumor growth in vivo, Figure S3: Synergistic effect of TMZ with APZ in U87MG, Figure S4: Combinational treatment of TMZ with APZ inhibits vascular invasion into glioma region, Figure S5: Peptide analysis to quantify volume of protein target candidates, Figure S6: RNA interference and recombinant Hsp70 transfection analysis.
